# Evaluation of a Prediction Protocol to Identify Potential Targets of Epigenetic Reprogramming by the Cancer Associated Epstein Barr Virus

**DOI:** 10.1371/journal.pone.0009443

**Published:** 2010-02-26

**Authors:** Kirsty Flower, Elizabeth Hellen, Melanie J. Newport, Susan Jones, Alison J. Sinclair

**Affiliations:** 1 School of Life Sciences, University of Sussex, Brighton, United Kingdom; 2 Brighton and Sussex Medical School, Brighton, United Kingdom; Karolinska Institutet, Sweden

## Abstract

**Background:**

Epstein Barr virus (EBV) infects the majority of the human population, causing fatal diseases in a small proportion in conjunction with environmental factors. Following primary infection, EBV remains latent in the memory B cell population for life. Recurrent reactivation of the virus occurs, probably due to activation of the memory B-lymphocytes, resulting in viral replication and re-infection of B-lymphocytes. Methylation of the viral DNA at CpG motifs leads to silencing of viral gene expression during latency. Zta, the key viral protein that mediates the latency/reactivation balance, interacts with methylated DNA. Zta is a transcription factor for both viral and host genes. A sub-set of its DNA binding sites (ZREs) contains a CpG motif, which is recognised in its methylated form. Detailed analysis of the promoter of the viral gene *BRLF1* revealed that interaction with a methylated CpG ZRE (RpZRE3) is key to overturning the epigenetic silencing of the gene.

**Methodology and Principal Findings:**

Here we question whether we can use this information to identify which host genes contain promoters with similar response elements. A computational search of human gene promoters identified 274 targets containing the 7-nucleotide RpZRE3 core element. DNA binding analysis of Zta with 17 of these targets revealed that the flanking context of the core element does not have a profound effect on the ability of Zta to interact with the methylated sites. A second juxtaposed ZRE was observed for one promoter. Zta was able to interact with this site, although co-occupancy with the RpZRE3 core element was not observed.

**Conclusions/Significance:**

This research demonstrates 274 human promoters have the potential to be regulated by Zta to overturn epigenetic silencing of gene expression during viral reactivation from latency.

## Introduction

Epstein Barr virus (EBV) infects and causes several diseases in humans including Burkitt's lymphoma, nasopharyngeal carcinoma, Hodgkin's disease, post-transplant lymphoproliferative disorder and infectious mononucleosis (glandular fever) [1]–[4]. Like other members of the gammaherpesviruses family, EBV infection persists for life following primary infection. The virus is maintained in a state of latency in memory B-lymphocytes and occasional reactivation and replication is considered to maintain the virus within individuals [5]. In EBV-induced lymphomas, EBV is also present in a latent state. In cell lines derived from these lymphomas, the viral genome is predominantly methylated [6]–[9]. The effect of silencing viral gene expression not only aids evasion from the immune system [10], but also prevents the destruction of tumour cells by viral replication. Indeed, reactivation of EBV from latency has been proposed as a route to treat EBV-associated lymphomas [11], [12].

EBV latency is disrupted following physiological activation of B-lymphocytes, through the expression of Zta (BZLF1, ZEBRA, EB1, Z) [13]–[15]. Zta is a sequence-specific DNA-binding protein, which resembles the bZIP family of transcription factors and plays a critical role in the reactivation of viral gene expression and replication of the genome. Through direct interaction with Zta response elements (ZREs) in promoters, Zta regulates the expression of viral and cellular genes. Many host and viral promoters that have been evaluated to date contain ZREs within the proximal five hundred nucleotides of 5′ sequence. Thus far, eight have experimentally verified binding sites for Zta in their proximal promoter regions:

*BSLF2+BMLF1* [16], [17]; *BRLF1* [18]; *BZLF1* [17], [19], [20]; the joint promoter for *BHLF1* and *BHRF1* [19]; the lytic *EBNA1* promoter Fp [21]; *BRRF1* [22]; and *BMRF1* [23]. Furthermore, 6 host genes are directly regulated by Zta through ZREs in their promoters *DHRS9* [*24*]; *EGR1* [25], [26]; *CIITA* [27]; *IL-8* [28]; *IL-10* [29]; and *IL-13* [*30*]. Zta interacts with a diverse range of ZREs [31] and multiple sites exist in some Zta-responsive promoters, sometimes in close proximity. One example is the viral promoter for the *BZLF1* gene, Zp, which contains the two functional ZIIIA and ZIIIB ZREs within a total span of 20 nucleotides [17], [19], [20].

Zta has the unusual feature of interacting with a sub-set of ZREs that contain a methylated CpG motif [32]. In some cases Zta is able to interact with the non-methylated ZRE, while for other ZREs the interaction with Zta is dependent on methylation [22], [26], [32]–[34]. This has led to the classification of ZREs into three classes: class I ZREs do not contain a CpG motif; class II ZREs contain a CpG motif that is recognized in both the methylated and non-methylated states; and class III ZREs contain a CpG motif but are only recognized when methylated [35]. The ability of Zta to interact with methylated CpG-containing ZREs allows Zta to activate gene expression in the latent viral genome despite the repressive methylation status and thus overturn the epigenetic silencing of the viral genome [22], [32]–[35].

To date, three genes with CpG-containing ZREs have been studied: EBV *BRLF1*, EBV *BRRF1* and human *EGR1* [22], [26], [32]–[35]. Of these, investigation of the regulation of the EBV gene *BRLF1* provided compelling evidence that the interaction of Zta with a methylated ZRE was instrumental in reactivating EBV into lytic cycle in B-lymphocytes [32]–[35]. The viral *BRLF1* gene includes three ZREs in the promoter proximal region [18], [32]. Two of the ZREs contain CpG motifs; RpZRE2, is a class II ZRE and the other, RpZRE3 is a class III ZRE [35]. The ability of a single point-mutation in Zta to differentiate between the interaction of Zta with methylated and non-methylated RpZRE3 allowed the relevance of the interaction of Zta with this promoter to be established [34], [36], [37]. The viral *BRRF1* gene contains two CpG-containing ZREs, which Zta only interacts with in their methylated states [22]. The presence of a CpG-containing ZRE in the promoter of the human *EGR1* gene, which is recognized by Zta in its methylated form, suggests that EBV may overturn epigenetic silencing of host genes in order to modulate the host cell environment [26].

The RpZRE3 core element (TCGCGAA), which was clearly shown to be required for overturning epigenetic silencing of *BRLF1* in B-lymphocytes [34], [36], [37], was chosen to identify all human genes that contain an exact match to this CpG-containing ZRE within the −500 to +1 region of the promoter and evaluate whether they are recognised by Zta in their natural context.

## Materials and Methods

### Identification of Human Promoters Containing RpZRE3-Core Elements: Initial Sampling

Promoter regions (defined as −500 to +1 base pairs from the transcription start site) of a sample of human protein-coding genes in Ensembl (49) [38] were extracted using the Biomart data management system [39]. The sample represented 40% of the human genome. A search was made for all occurrences of an exact (forwards and reverse) match to identify those promoters with the TCGCGAA RpZRE3 core element.

### Identification of Human Promoters Containing RpZRE3-Core Elements: Whole Genome Scanning

An identical screen was undertaken on the entire human genome from Ensembl (50) [40], resulting in the identification of 274 promoters which were designated the RpZRE3-promoter-274 data-set. The 7 nucleotides of the RpZRE3 core element, along with 10 flanking nucleotides on each side, were extracted from each of the 5 genes from the initial sampling. The Transcription Factor Binding Site (TFBS) Perl modules [41] were used to create a Position Weight Matrix (PWM) based on the entire length of these 27-nucleotide sequences. TFBS Perl modules were also used to search the promoter regions (−500 to +1) of all human protein coding genes in Ensembl 50 [40] which were extracted using Biomart [39]. Those promoters that matched the PWM with a threshold value >80% and contained an exact match to the RpZRE3-core element were additionally filtered using 2 criteria (a) the presence of CpG islands and (b) function (based on the over-representation of Gene Ontology (GO) terms [42]). The location of CpG islands were predicted using the EMBOSS program CpGPlot [43] with default options. Only genes with a CpG island present in the promoter were retained. The GO term annotations for molecular function and biological processes were extracted from Ensembl for each gene [40]. The number of genes in the RpZRE3 promoter dataset with each GO term annotation was compared with the total number of genes in Ensembl with the same GO term. The GO terms that occurred with a significantly higher frequency (p<0.05) in the RpZRE3 dataset, compared to entire genome, were defined as over-represented.

### DNA Binding Analysis by EMSA on RpZRE3 Containing Promoters

Double stranded labelled DNA probes were made by labelling 6 pmol of oligonucleotide (27 nt long) at the 5′ end with [γ-^32^P]ATP (30 µCi) using polynucleotide kinase (Roche). 12 pmol of the complementary oligonucleotide strand was added and incubated with the labelled single strand at 95°C for 2 minutes, 65°C for 10 minutes, and 37°C for 30 minutes to anneal the strands. The concentration of the probe was 33.3 nM. The oligonucleotides comprised the core 7-mer sequence surrounded by 20 nucleotides corresponding to the cognate flanking sequence for each promoter and were synthesised. Where indicated in the figure, the central CpG motif was methylated on both cytosines during synthesis (Sigma).

Zta protein was *in vitro* translated using wheatgerm extract (Promega). This was incubated with the labelled probe (at a final probe concentration of 3.3 nM) for 30 minutes at room temperature, before the sample was fractionated on an 8% native polyacrylamide gel at 100 volts for 1 hour. Following detection of the radio labelled DNA using a Storm phosphorimager, the relative signals were quantitated using ImageQuant software (GE Healthcare, UK).

Competition EMSAs were carried out with a 100X excess of a double stranded non-labelled oligonucleotide in addition to the standard EMSA reaction. Equal quantities of each of the complementary oligonucleotides were annealed in the same manner as the labelled probes and diluted to yield a solution concentration of 3.33 µM. This was added to the EMSA reaction at a final concentration of 333 nM (i.e. 100X excess).

### Oligonucleotides

The oligonculeotides used were double strand versions of the following sequences (5′-3′):

XPC GGTGCGTCACTCGCGAAGTGGAATTTG

ZC3H8 GCTTCCCGGCTCGCGAAAGGGAGGACC

HDAC2 TCCCCCACTGTCGCGAAGCTCCCGCCC

MNT CCGCGGCGTCTCGCGAAGGGAGGGGCG

Cyclin L2 GGGCGGCTCCTCGCGAAGCTCCACGGC

RpZRE3 GTTTATAGCATCGCGAATTTTGAGTGC

CAPN2 CCGGGGAGGCTCGCGAATCGCGGTCCA

CDO1 CGTCCCAGCGTCGCGAACCACAGCGGC

FALZ GGCGCGCAGCTCGCGAAATGCCCGGCG

KIF1B GCTTCGGCCCTCGCGAAACTCCGCCCG

LLGL1 TCGGCCGGGCTCGCGAAGGGACGCCCG

LMO4 GGATCCCGGGTCGCGAAGGGCAGCCCA

MBD4 CCTCCTGCTCTTCGCGAACCGCCCCGC

PLEKHJ1 AGCCGCTCCCTCGCGAAAGTTGGCCCC

PRKD1 CTTCCTGGGGTCGCGAACTTCCCGGGC

SEC14L CGCCCGCTACTCGCGAAGCCCAGCCCG

TADA3L GCTGCGCTTCTCGCGAAAGGGCAGGCA

TOP2B CCGCGCCCCATCGCGAAGATCCGGAGC

XPCLMNTR GGTGCGTCACTCGCGAAGGGAGGGGCG

MNTLXPCR CCGCGGCGTCTCGCGAAGTGGAATTTG

XPC Mcore GGTGCGTCACCCCCTTAGTGGAATTTG

XPCLM3Mcore GGTCCGCCTCCCCCTTAGTGGAATTTG

AP1 mut GATCCACCCCTTAGAGGAAAACATACG

### Prediction of Transcription Factor Binding Sites Using Promo

The transcription factor binding site prediction program PROMO [44] was used with the default setting of 15% dissimilarity value, to identify potential bZIP transcription factor binding sites within the XPC oligonucleotide.

## Results

### Identification of Human Promoters Containing the RpZRE3-Core Element: Initial Sampling

The initial search for RpZRE3-core elements in a sample of human promoters revealed 67 genes with an RpZRE3-core element. 5 of these that are involved in gene regulation were selected for DNA binding analysis. These 5 genes were ZC3H8 (ENSG00000144161), HDAC2 (ENSG00000196591), XPC (ENSG00000154767), MNT (ENSG00000070444) and CyclinL2 (ENSG00000116148) and together with the RpZRE3 element from the viral *BRLF1* promoter (Rp), formed the RpZRE3-promoter-6 data-set.

**Figure 1:**
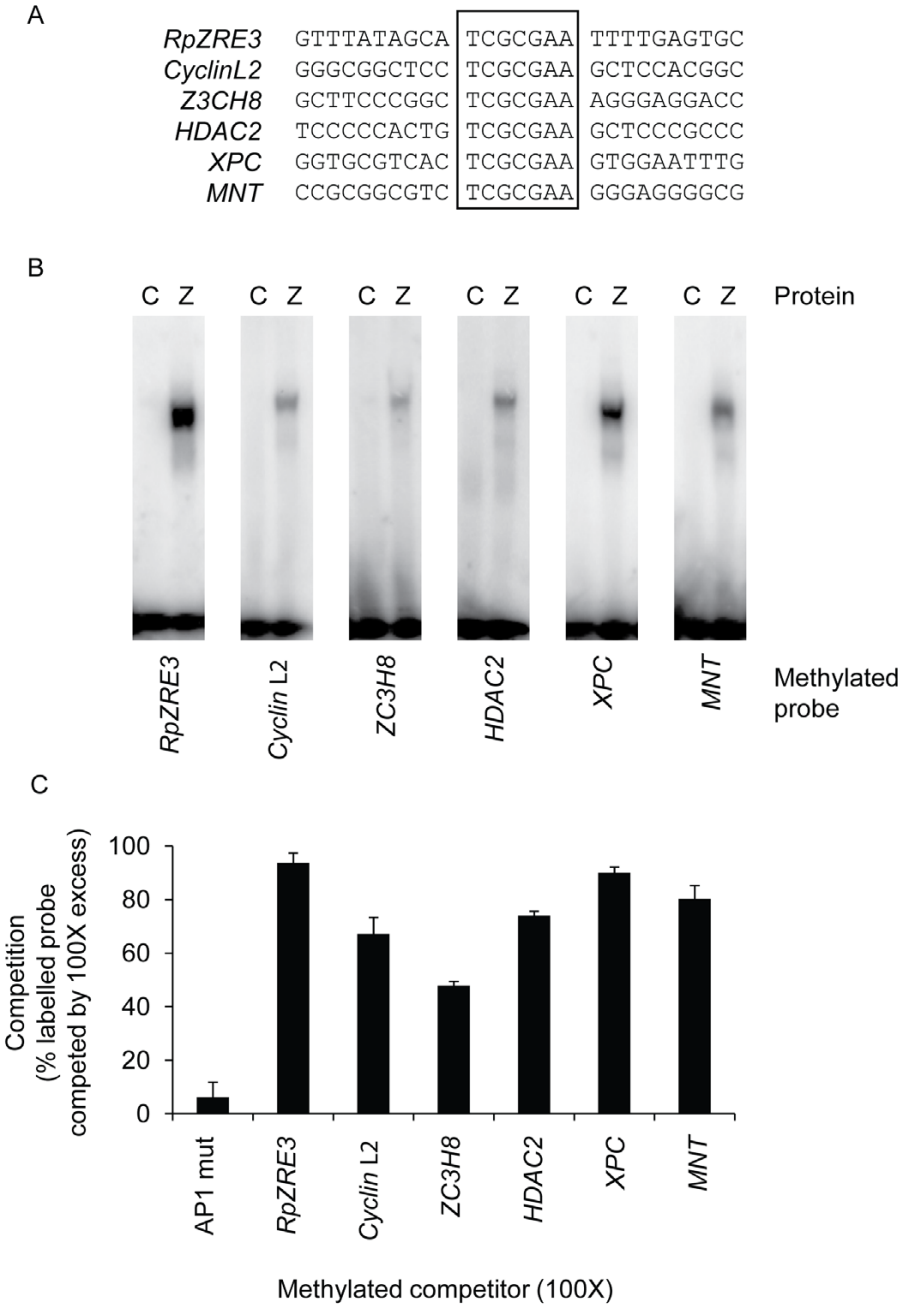
Interaction of Zta with methylated RpZRE3 core element from 5 human promoters. (a) The nucleotide sequence of one strand of each oligonucleotide spanning the indicated ZREs is shown, with the conserved RpZRE3 core element (TCGCGAA) aligned. Double strand versions of these sequences were used as probes in EMSA, with the 2 cytosines within the CpG core motif methylated. The RpZRE3 from EBV BRLF1 promoter is included. (b) DNA binding between *in vitro* translated Zta with each probe was undertaken by EMSA. The ability of Zta (Z) to interact with the probe was compared with an unprogrammed translation lysate (C) as a negative control. The complex was separated on an 8% gel by electrophoresis and visualised by phosphoimaging. The excess probe can be seen at the bottom of the gel. The probe used in each experiment is indicated below the gel. (c) Competition of each ZRE sequence (at 100X excess) against labelled RpZRE3 was determined by competition EMSAs. Data from at least 2 experiments was taken to calculate competition i.e. the percentage of labelled probe displaced by unlabelled competitor. Methylated ZREs were used for both probe and competition. AP1 mut, an oligonucleotide previously shown not to interact with Zta [36], was used as a negative control to define the level of non-specific binding. Error bars indicate standard error.

DNA-binding assays were undertaken with the methylated forms of each of the 5 human promoters using 27mer double strand oligonucleotides encompassing the 7-nucleotide core element and 10-nucleotide flanking region on each side together with RpZRE3 from Rp. Electrophoretic mobility shift assays (EMSA) were undertaken with *in vitro* translated Zta protein. Zta protein/DNA complexes formed readily with the oligonucleotides from all six promoters (Figure 1). The specificity of the assay is shown by the lack of complex formation with control protein. In addition, we undertook competition experiments with an excess of unlabelled oligonucleotides and included a version with a mutant ZRE to further probe the specificity of the interaction. This confirms that Zta interacts with all six RpZRE3 core elements specifically.

### Identification of Human Promoters Containing RpZRE3-Core Elements: Whole Genome Scanning

**Figure 2:**
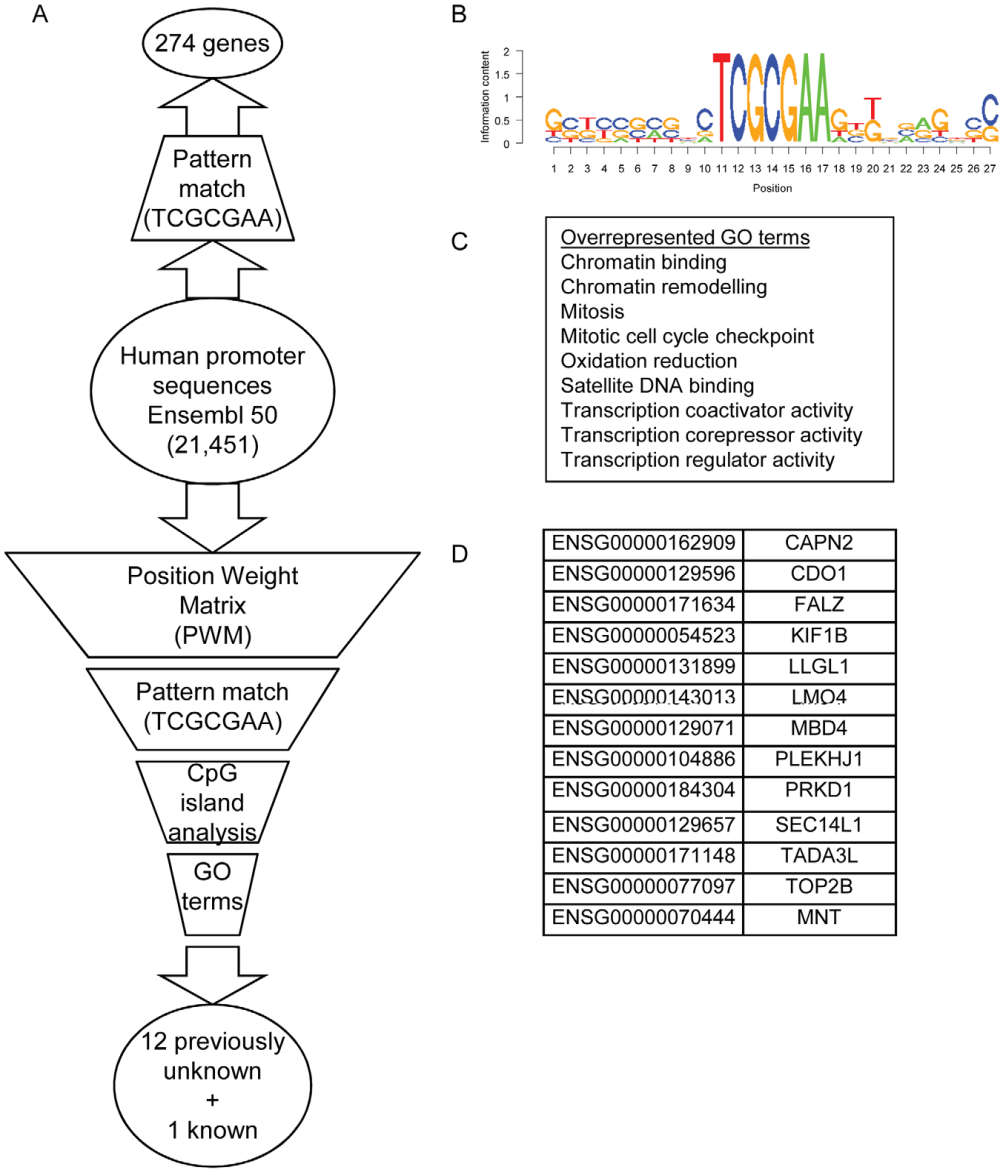
Identification of human promoters containing RpZRE3 core elements in the human genome. (a) The bioinformatics analysis undertaken for the genome wide scan is represented as a flow diagram. Genetic input or output is represented by ovals, and filters by trapeziums. (b) A Position Weight Matrix (PWM) was created using the aligned sequences of the RpZRE3-promoter-6 data-set. These sequences are shown in Fig. 1(a). This was used to filter the genomic data from the human promoter sequences (−500 to +1). (c) Overrepresented GO terms found for the 274 genes were identified. (d) The 13 genes identified as candidate genes, 12 previously unknown genes and 1 from the initial screen, and their associated Ensembl accession codes.

The complete human genome was scanned for additional RpZRE3-core elements. This resulted in a data-set of 274 genes, denoted the RpZRE3-promoter-274 data-set (see Table S1). To assess whether there was an influence of flanking sequence on the interaction of Zta with these ZREs we undertook a systematic filtering process. The RpZRE3-promoter-274 dataset was first filtered by a Position Weight Matrix (PWM), which was generated from the RpZRE3-promoter-6 data-set. Matches were further filtered for genes with at least one CpG island in the promoter and an over-represented GO term. This resulted in 12 previously unidentified promoters and a further one that had been identified in the initial screen (Figure 2). Together with the promoters from the initial screen and the viral *BRLF1* promoter (RpZRE3-promoter-6 data-set), these form the RpZRE3-promoter-18 data-set.

**Figure 3:**
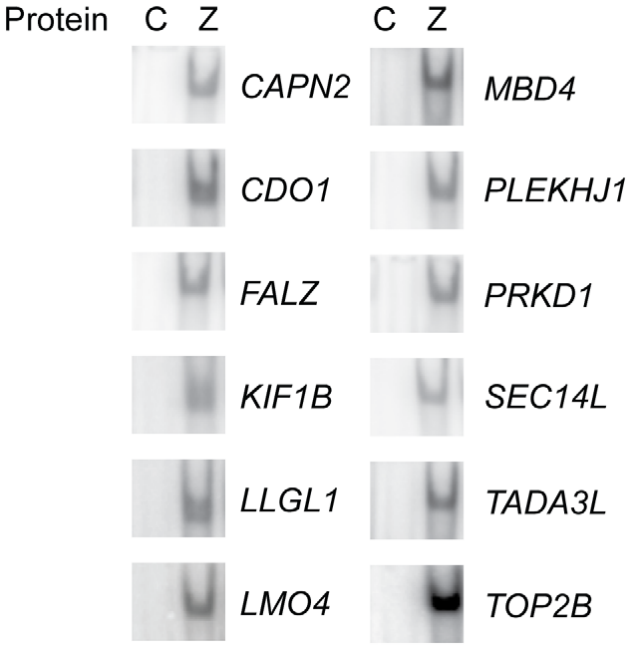
Interaction of Zta with methylated RpZRE3s found in human promoters. EMSA analysis with *in vitro* translated Zta protein (Z) or an unprogrammed translation lysate (C) with the probes indicated to the right of each gel was carried out as described in Fig. 1.

Oligonucleotides were designed using the same principles with 10 nucleotides of flanking sequence on each side of the RpZRE3-core element, and the ability of Zta to interact with each site in its methylated form was assessed. The EMSA analysis revealed that all twelve of the newly identified methylated promoters were recognised by Zta (Figure 3).

**Figure 4:**
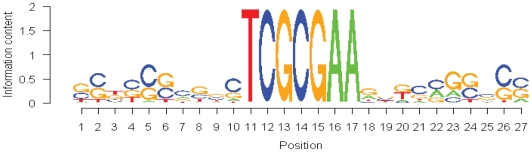
Analysis of the RpZRE3 core element flanking sequence. (a) A Position Weight Matrix (PWM), created using the probe sequences of the dataset RpZRE3-promoter-18.

This analysis demonstrated that for the RpZRE3-promoter-18 data-set, the flanking sequence surrounding the methylated core 7-mer element did not have a profound effect on the ability of Zta to interact with promoters and therefore the core element was sufficient to facilitate binding. This was illustrated by the generation of a PWM using sequence from the RpZRE3-promoter-18 data-set (Figure 4), which revealed negligible sequence conservation outside of the core element.

### Recognition of the Non-Methylated Promoters

Zta is able to recognise many response elements which do not contain a CpG motif and therefore do not have a methylated core element (Class I ZREs) [15], [35]. In addition, Zta recognises one CpG containing ZRE, RpZRE2, even in the absence of methylation (Class II ZREs) [33]. The ability of Zta to recognise the promoters in their non-methylated forms may impact on the ability of EBV to alter their gene expression, so we investigated the classification of ZRE for each of the 17 human promoters.

**Figure 5:**
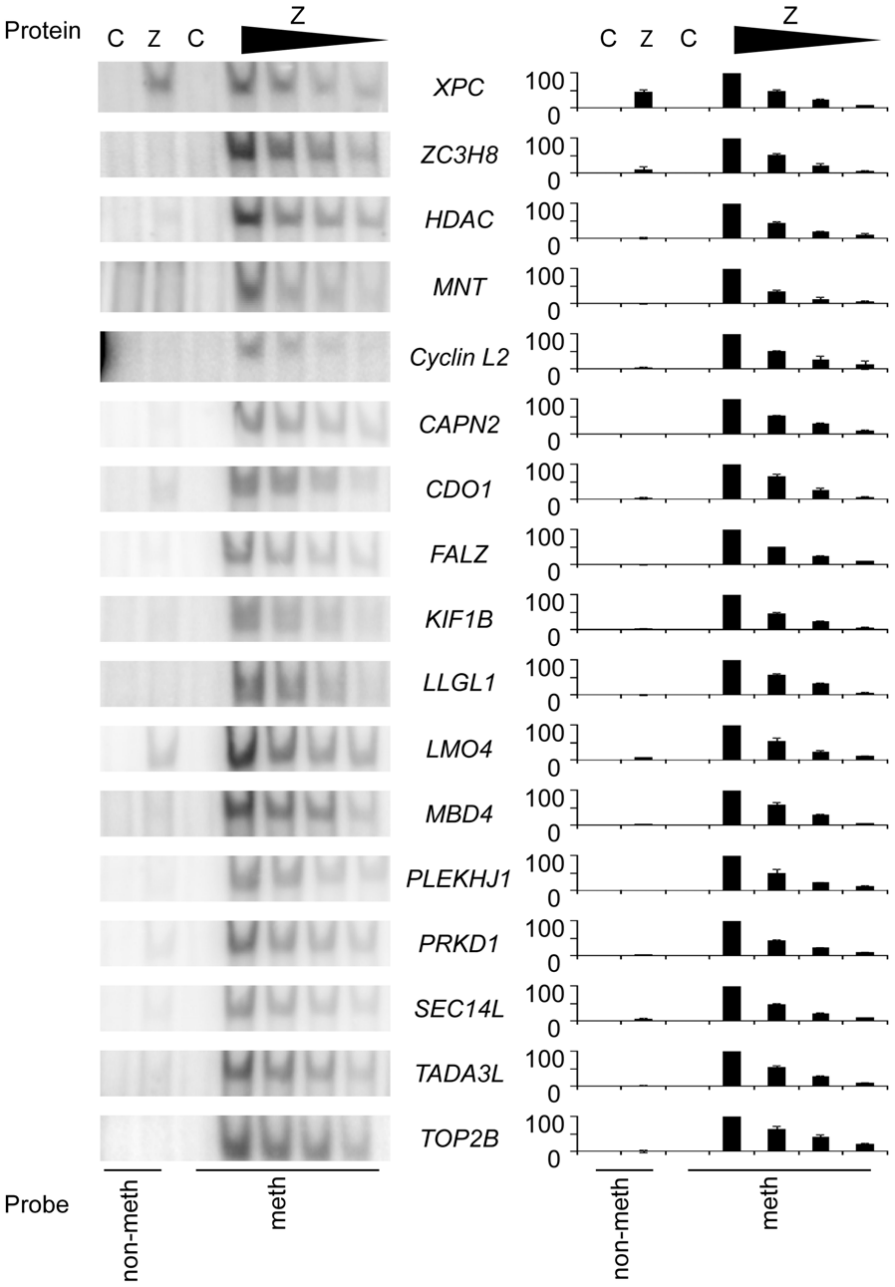
Comparison of Zta interaction with non-methylated and methylated RpZRE3s from human promoters. DNA binding analysis of *in vitro* translated Zta protein (Z) or an unprogrammed translation lysate (C) with both non-methylated and methylated versions of the probes indicated to the right of each gel was carried out by EMSA as described in Fig. 1. A titration of Zta protein (1∶2, 1∶4, 1∶8) was used to compare non-methylated binding to that of the methylated equivalent. Quantitation of the complexes formed between Zta and the non-methylated and methylated probes, from at least 2 experiments, is represented as a histogram to the right of the corresponding gel. Complex formation is shown relative to the maximum binding for each probe. Error bars indicate standard error.

A series of DNA binding experiments with the oligonucleotides representing the human promoters in the RpZRE3-promoter-18 data-set, were undertaken comparing non-methylated with methylated RpZRE3-core elements. The interaction of Zta with the sites was quantitative, as demonstrated by the reduction in complex formation as the Zta protein concentration was titrated on each of the methylated promoters (Figure 5). All bar one display negligible binding to the non-methylated oligonucleotides (at least 10-fold lower than to the methylated sites) and can therefore be classified as class III ZREs.

Zta displayed a reproducible interaction with the non-methylated XPC oligonucleotide; the interaction reached 50% of the binding observed with the methylated oligonucleotide (Figure 5). This raises the possibility that the XPC core RpZRE3 element is influenced by the flanking sequence to behave as a class II ZRE.

### Additional ZRE Juxtaposed with an RpZRE3-Core Element in Flanking Region

**Figure 6:**
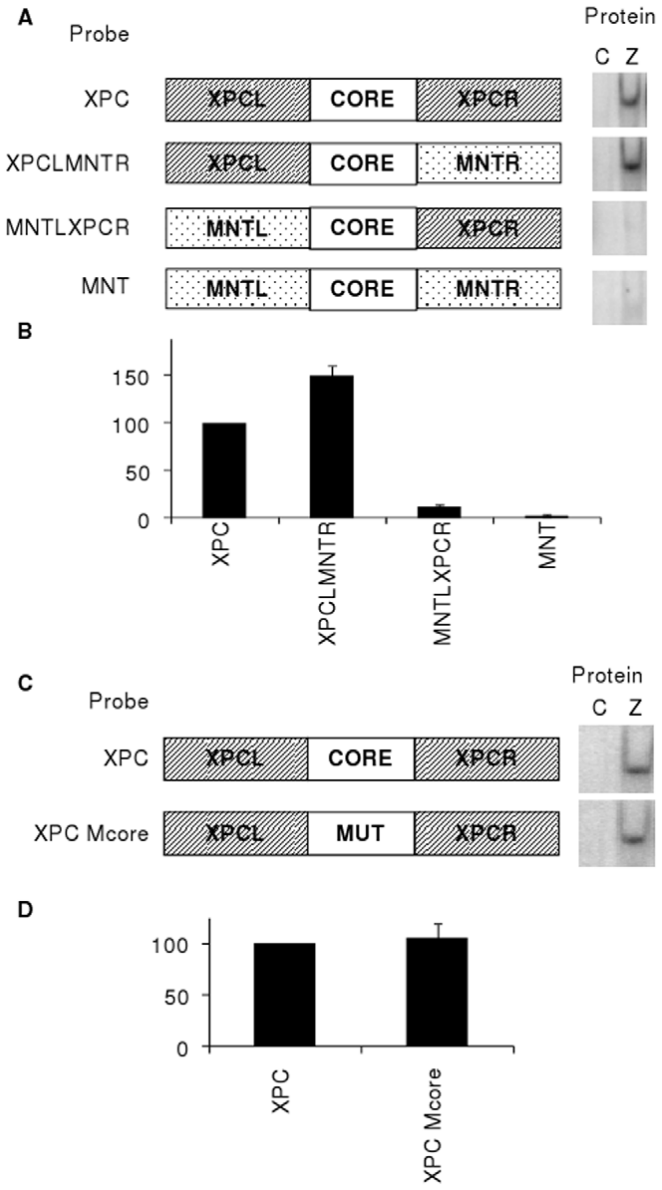
The effect of flanking region upon Zta binding to non-methylated XPC probe. (a) Schematic representation of the probes, illustrating the XPC and MNT probes, and the flank swap probes XPCLMNTR and MNTLXPCR, adjacent to the corresponding EMSA analysis, carried out in the same manner as described in Fig. 1. (b) Quantification of the complexes formed with each probe, from 2 experiments was undertaken. Complex formation is represented as a percentage of the Zta complex with the non-methylated XPC probe. Error bars indicate standard error. (c) Schematic representation of probes, indicating mutated core sequence. EMSA analysis was carried out as described in Fig. 1. (d) Quantitation of the complexes formed with each probe, from 2 experiments was undertaken. Complex formation is represented as a percentage of the Zta complex formed with the non-methylated XPC probe. Error bars indicate standard error.

To identify whether sequences conferring methylation independent recognition was confined to a specific flank of XPC, a series of mutant oligonucleotide probes that exchanged flanking sequences between XPC and a class III site (MNT), that is not recognised when non-methylated, were designed. Analysis of the interaction with Zta by EMSA revealed that the ability to confer Zta binding resided within the 5′ sequence of XPC; the hybrid XPCLMNTR was able to bind but MNTLXPCR was not (Figure 6). This suggested that the 5′ XPC flanking sequence of the RpZRE3 element influenced binding. However, it was surprising to discover that mutation of the RpZRE3 element within XPC did not prevent interaction with Zta (Figure 6).

**Figure 7:**
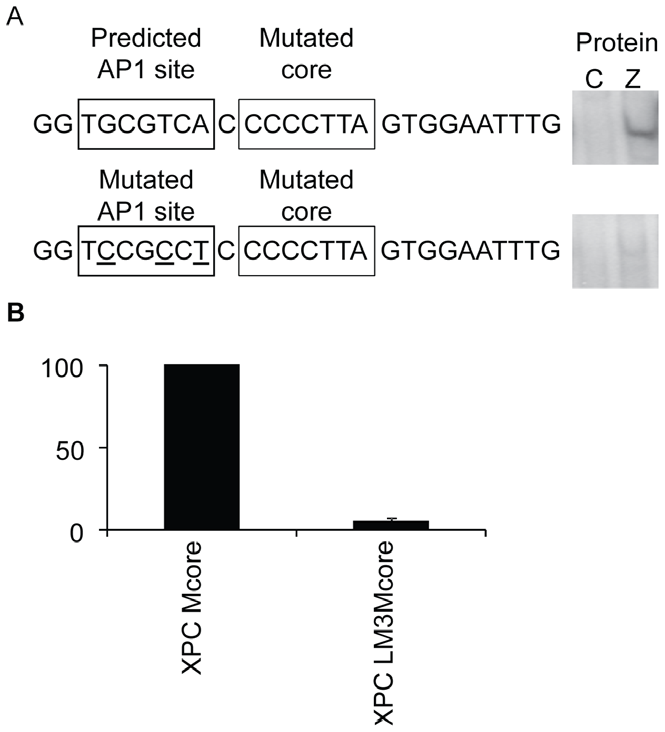
Uncovering an additional ZRE in the 5′ flank of the XPC probe. (a) PROMO transcription factor binding site prediction software identified an AP1 site which is highlighted in the 5′ flank sequence of XPC. The specific nucleotide mutations are underlined. The complexes formed by EMSA are shown to the right of the corresponding probe sequence. (b) Quantitation of the complexes formed with each probe, from 2 experiments, was undertaken. Complex formation is represented as a percentage of the Zta complex formed with the XPC mutated core probe. Error bars indicate standard error.

A further explanation for the interaction of Zta with the non-methylated XPC promoter is that an obscure ZRE is present in the 5′ flank. To address this further we attempted to identify putative ZREs in the XPC promoter using the transcription factor binding site prediction program PROMO [44], [45]. Although this program contains a PWM for Zta binding sites, none were predicted in this sequence. However, PROMO predicted the presence of AP1, c-fos and c-jun binding sites within the 5′ flank of XPC (5′TGCGTCA) (Figure 7). c-fos and c-jun proteins are both members of the bZIP transcription factor family; they form AP1 DNA binding activity as either homodimers or heterodimers. It is relevant that fos/jun dimers share some DNA recognition motifs with Zta [15]–[17], [31], [46]–[49]. This led to the hypothesis that the predicted AP1 site in the 5′ flanking sequence is an additional ZRE that is responsible for binding to the non-methylated XPC oligonucleotide. To test the hypothesis, further mutations were introduced into the 5′ XPC flanking sequence in the predicted AP1 site (5′TCCGCCT). The ability of Zta to interact with the double AP1/core mutant site (XPCLM3Mcore) was compared with the core-only mutant. Dual mutation of the predicted AP1 site and the core abrogated the ability of Zta to interact with the XPC oligonucleotide, demonstrating that binding of Zta to the non-methylated XPC site resided with this AP1 site (Figure 7).

This analysis showed that the RpZRE3 core element is not recognised in its non-methylated state in the context of any of the viral or cellular promoters in the RpZRE3-promoter-18 data-set, and that this ZRE is specifically recognised when methylated.

## Discussion

A computational search revealed a set of 274 human genes with cellular promoters containing the 7-mer RpZRE3 core element. Flanking sequence can influence the interaction of some transcription factors with DNA, for example, the interaction of the E2F family with DNA is influenced by a region of at least 8 nucleotides 5′ and 11 nucleotides 3′ to the central nucleotide [50]. Before assigning all 274 genes as candidates for regulation by Zta, it was important to assess whether the cognate flanking sequence had a profound effect on binding by Zta. Consideration of the sequences represented in the flanking sequence of this set of genes revealed it to be diverse; all four nucleotides were represented in 55% of positions and three of the four nucleotides were represented at the remaining positions. Indeed, the immediate flank of the RpZRE3 core element, consisting of four nucleotides both 5′ and 3′, had 100% representation of all four nucleotides. It can therefore be concluded that the flanking sequence does not prevent binding to the methylated RpZRE3 core, and a PWM generated from the RpZRE3-promoter-18 data-set showed little conservation outside the core motif. In contrast, the interaction with the non-methylated core element initially appeared to be influenced by flanking sequence in only 1 of the 18 promoters. However, further analysis revealed this to be due to the presence of a second ZRE in the flanking sequence.

These results show that the approach of undertaking a computational pattern match search for the 7-mer RpZRE3 core element is a fast and reliable method to identify promoters containing ZREs that are recognized by Zta only when they are methylated (class III ZREs).

The identification of two adjacent juxtaposed ZREs in the XPC promoter presents an interesting problem; can both sites be occupied at once? The natural juxtaposition of ZREs has been previously described for a viral promoter; *BZLF1* and a cellular promoter *EGR1*. In the *BZLF1* promoter, the elements are situated with 12 base pairs between the central nucleotides; both can be occupied simultaneously and both are functionally relevant [17], [19]–[20]. For the *EGR1* promoter, the elements are immediately adjacent with just 8 nucleotides between the central nucleotides [25], [26]. There is no evidence for simultaneous occupation by Zta and only one element appears to be functional *in vivo* [26]. The arrangement of the *XPC* promoter places the elements 8 nucleotides apart, identical to *EGR1*, and there is no evidence from the DNA-binding experiments for simultaneous occupation of the sites. As one ZRE is recognised in a methylation-dependent manner and the other is recognised when non-methylated, it is possible that the arrangement may provide a fail-safe mechanism to ensure that the gene is regulated in both its methylated and non-methylated states.

The simple computational approach taken to identify the location of this methylation-dependent and novel DNA-binding motif in human gene promoters identified 274 genes potentially regulated by overcoming epigenetic silencing during the viral replicative cycle. Given the known functions of Zta in reprogramming viral gene expression, disrupting cell cycle control and replicating viral DNA, it is interesting to note that the Gene Ontology terms that are over-represented in the RpZRE3-promoter-274 gene-set are largely involved in transcription, chromatin re-modelling and mitosis. This strongly suggests that Zta may activate this set of cellular genes in order to accomplish these functions. Testing whether these genes are activated during latency disruption in memory B-lymphocytes *in vivo* is technically challenging given both the scarcity of memory B-lymphocytes in peripheral circulation and the infrequency of latency disruption *in vivo*.

The co-location of the RpZRE3 core element in 274 human promoters is unlikely to have been driven by an evolutionary advantage to the virus, but may reflect the involvement of a cellular transcription factor interacting with the same element. This would suggest that this set of genes share a common mode of regulation during human development or differentiation. Furthermore, it will be interesting to question whether co-occurrence of the RpZRE3-core element with other transcription factor binding sites forming a co-operative *cis*-regulator module which may illuminate the regulation of these host and viral genes.

## Supporting Information

Table S1 RpZRE3-promoter-274 data-set: A table of genes which contain a RpZRE3 core element in the 500 bp promoter region.
(0.05 MB XLS)
